# Keeping calm on a busy day—an interpersonal skill home care patients desire in health workers: hermeneutical phenomenological method

**DOI:** 10.1186/s12912-022-00825-1

**Published:** 2022-02-25

**Authors:** Siw Watz, Kari Ingstad

**Affiliations:** 1 Oslo Metropolitan University, Karethe Johnsens hus, Kunnskapsveien 55, 2007 Kjeller, Norway; 2 University Nord, Pb. 93, 7601 Levanger, Norway

**Keywords:** Home care service, Interpersonal relations, Nurse-patient relations, Patient perspectives

## Abstract

**Background:**

In Western countries, many health and social care provisions have been transferred to primary care, and most older patients wish to remain in their own homes for as long as possible. For older patients who live alone, health workers could be their only personal contacts. Hence, health workers’ personal skills affect their relationships with these patients. Accordingly, this study aimed to shed light on the interpersonal skills needed by health workers to establish good relationships with older home care patients and highlight the importance of interpersonal skills training in nursing education.

**Methods:**

This study adopted a hermeneutical phenomenological approach. The qualitative method was used to elicit data on patients’ perspectives. Ten home care patients were interviewed individually in their own homes between December 2019 and January 2020.

**Results:**

Despite individual variations, health workers’ interpersonal skills are of significance with regard to the social well-being of patients living at home. The findings revealed that patients want health workers to be mentally present, congruent in their communications, calm and relaxed during the available time spent with them, and capable of facilitating autonomy.

**Conclusions:**

It is important to present patients’ perspectives to ensure that nursing education is geared towards patients’ best interests.

**Trial registration number:**

The Norwegian Centre for Research Data (NSD): 953937.

## Background

In present-day healthcare, in line with a greater focus on person-centred care (PCC), eliciting patients’ perspectives on the services they receive is gaining increasing importance [1, 2]. One of the pillars of modern healthcare is the voice of patients, also seen as their ‘narrative’ [3]. Building care around the needs of individuals and knowing patients as unique human beings are the cornerstones of the PCC philosophy [4]. When PCC is emphasised in clinician-patient relationships, patients’ physical health and resources as well as their emotional, mental, and social well-being will be enhanced [5, 6]. Care practices that can improve patients’ lives and well-being should be at the centre of home care services (HCS) [7] through PCC and autonomy. Over the last decade, healthcare policies in the Western world have seen a shift towards autonomy in care [8], which is highly valued [9].

In recent years, the number of patients transferred to primary care in Western countries has increased. However, the average length of hospital stay has reduced, with the result that primary care now has more severely ill patients, who need more complex care and treatment [10]. Since most seriously ill patients still want to live at home, in the comfort of a familiar environment [11, 12], there is a need for more advanced knowledge and skills among home care staff [10].

The HCS are multi-professional services, and aim to provide coordinated healthcare for patients in their homes, including practical assistance as well as home care nursing [13]. Therefore, HCS are playing an increasingly significant role in Western healthcare [14]. In Nordic countries, care services, which are well developed, are provided by the public sector [15]; the welfare state is responsible for the entire population’s HCS [16]. In Norway, local authorities have to decide on an individual basis whether patients are to receive HCS by taking into account the nature of the services, how extensive they are, and how well they are organised [17]. In recent decades, HCS have increasingly been subject to organisational, structural, and financial guidelines, which means that each patient visit is carefully planned in terms of the care to be provided and the availability of time [15].

Although the home setting is not designed for caregiving [8], in HCS, that is where patients receive care. Studies on patients’ perceptions of home care nursing conducted both in Scandinavia and other European regions [18] generally report a high level of satisfaction with care services. Patients’ statements relating to emotional support as well as relief of distress and anxiety have indicated the quality of care [19]. However, some studies have indicated a low level of satisfaction, which could be explained by the lack of autonomy among patients and inadequate provision of psychological care by their caregivers [18].

Despite the fact that older patients living at home mostly receive treatment and care in the community, data on relational needs from their viewpoint are limited [9, 20]. Since some studies have examined the topic from the perspective of professionals and family members, rather than the older persons themselves [12], we decided to base our study on the perspectives of older patients and shed light on the interpersonal skills they would like health workers to possess.

### Interpersonal skills

Interpersonal skills are relational and process-oriented [21]. The nurse-patient relationship is an interpersonal process that develops over time, as well as a professional relationship with a structure that implies that ‘patients need help and nurses have the knowledge to provide such help’ [22]. Peplau [22] aims that the interactional phenomenon that occurs in the relationship between patients and nurses is necessary for regaining health and wellbeing. According to Peplau, patients seek sympathy, respect and regard for personal dignity. They want their nurse to be interested in them as persons and they want to be heard. In addition to respect, important elements of interpersonal skills include: being attentive to patients with open verbal, non-verbal, and intuitive communication; being personally present with them in the moment; and having a caring intent and interest in their ideas, concerns, and needs [21].

In the patient-health worker relationship, some movements between them will always take place in the intersubjective field—an area shared by two or more people, which contains feelings, thoughts, and knowledge, as well as provides information about the nature of the relationship [23]. The intersubjective bonds between people make them feel that the world is social, interpersonal, and relational [24]. The mutual influence between care providers and care recipients indicates that healthcare is relational [25], and encounters between them will reveal the health workers’ interpersonal skills, which are crucial in creating positive moments and well-being for patients [13].

In Norway, the time available for each patient visit is determined by legislation. Hence, the duration of an interaction between a health worker and a patient can range from a few minutes to several hours. Even when time is limited, the moment could still be important given that mutual actions and intersubjective contacts could be realised in an instant, suddenly changing the relationship [26]. If verbal or non-verbal communication makes patients realise that health workers understand their situation, it gives meaning to the relationship. In the health worker-patient relationship, through a nod of the head with eye contact, the former can convey to the latter ‘I understand how you feel’, and when this takes place in a ‘moment of meeting’, it can lead to change, greater mutual understanding, trust, and a feeling of genuineness [23], which can be referred to as congruence—a holistic correspondence between verbal and non-verbal communication.

People have inherent needs to be in relationships with others, master various activities, and make their own decisions [27]. Such needs, which are internal and shared by all people, have been described as basic psychological needs for relatedness, competence, and autonomy [28], and their satisfaction presupposes support from others, which is called ‘autonomy support’ [27]. This could mean that health workers possess interpersonal skills, such as offering choices to patients, wherever possible, because a key aspect of autonomy orientation is the ‘experience of choice’ [28] and caregivers ‘ought to leave value-laden decisions to their patients’ [29].

### Aim

This study explored older home care patients’ perspectives on their relationships with health workers, which gave rise to the following research question:

- **What interpersonal skills do health workers need to establish a good relationship with older home care patients?**

## Methods

This study adopted a phenomenological hermeneutic approach, which involved eliciting patients’ experiences regarding their relationships with health workers, with the aim of gaining access to people’s common-sense thinking and interpreting their actions, behaviours, and social world from their point of view [30].

### Recruitment, sample, and procedure

The data for this study were derived from individual interviews with 10 patients (average age 80 years; nine women and one man), who lived alone, and had collectively experienced around 45,000 visits to their homes. They were selected by the HCS leaders in two local authorities (one rural and one urban), based on the following inclusion criteria: recipients of HCS for at least 2 years, alert and aware of the time and place, and able to provide consent and willingness to share their experiences. All the participants were both verbaly and written informed about the study. The participants are presented in Table 2. All names are pseudonyms.

While the participants had different diagnoses and assistance needs, they most commonly needed help with medication, wound care, cooking, and personal hygiene.

### Data collection

A semi-structured interview guide with an exploratory design formed the basis of the interviews, which lasted 45 to 60 min, and were conducted between December 2019 and January 2020 in the patients’ homes, according to their wishes for reasons of mobility. The researcher was keen to adopt a humble, pleasant, and respectful demeanour in the interviews, so as to inspire confidence and trust [31]. The interviews were audio recorded, with the consent of the participants, and transcribed after each interview by the first author. The stepwise process is explained in Table 1.

**Table 1:** Stepwise process from start to analysis

Step 1	Step 2	Step 3	Step 4	Step 5	Step 6	Step 7
Writing the interview guide	Application and approval, Norwegian Centre for Research Data	First contact and pre-meeting with leader and trainee	Obtaining consent for participation from patients	Interviews conducted in patients’ homes	Listening to and transcribing recordings	Categorisation, coding, analysis

The participants were talkative. They were pleased to be asked questions and to have the opportunity to talk about their experiences.

### Data analysis

The data analysis was conducted using manifest content analysis [30], with a hermeneutical phenomenological approach. The analysis commenced during the interviews, with some findings being noted down, leading to follow-up questions. Subsequently, the data was prepared for analysis [30] and continued with repeated readings of the transcribed material. This first naive understanding of the text provided an opportunity to reflect on the data’s overall meaning. In this process, the data were segmented into smaller parts and labelled, and then placed into different categories. To create meaning out of the interviews, the data were analysed back and forth, where the transcribed interviews were read and picked apart a majority of times. The second author observed the data analysis process and contributed inputs. To strength the analysis process, the categories were adjusted and changed after dialogue between the authors. The purpose of coding and categorizing was to achieve a further understanding of the collected data, by studying the segmented sentences and categories. This process is consistent with the hermeneutic circle, as described by Heidegger [32].

### Rigor/trustworthiness

Rigor or trustworthiness was established via four criteria: credibility, dependability, confirmability and transferability [33]. The researcher’s strategies to guarantee credibility are prolonged engagement, persistent observation and investigator triangulation [34]. The first author, who performed the interviews, was an experienced nurse. She was familiar with the setting and context, which is necessary to build trust and to collect rich data. Persistent observation involves identifying characteristics and elements that are most relevant to the problem or issue under the study [34]. The analytic process involved, examines the text as a whole, examines its constituent parts, and then returns to the text as a whole. This is referred to as the hermeneutic spiral or hermeneutic circle [32]. Coding, analysis and interpretation decisions were performed by two researchers, which ensured investigator triangulation. In this process of comparing the parts to the whole, the pursuit of understanding became “deeper and deeper” and “richer and richer”. This process also verified that findings were consistent with the raw data, and ensured dependability. To achieve a set of information for confirmability, data regarding both the context and participants’ behaviours and experiences, were described, instead of only describing participants’ behaviours and experiences. This approach makes the data meaningful for a reader or an outsider and ensures transferability.

## Results

In this section we first describe the participants characteristics. Next we outline the codes and categories our analysis revealed for the interpersonal skills that home care patients would like to see in health workers, thus shedding light on areas where there is scope for improvement.

### Participants characteristics

The participants characteristics are presented in Table 2:

**Table 2:** Database

Name	Rural/Urban	Age	Care needs	No. of daily visits
Gunn	Urban	86	Getting up, going to bed, dressing, meals	3x
Kristin	Urban	82	Medication, meals, showering	3x
Thea	Urban	63	Getting up, showering, wound care	2x
Anne	Urban	76	Personal care, showering, meals, toileting	6x
Tale	Urban	84	Morning care, wound care, meals	4x
Kåre	Urban	87	Medication, putting on stockings, meals	2x
Gerd	Rural	75	Putting on stockings, putting to bed, showering	3x
Grete	Rural	83	Medication, showering	2x
Trine	Rural	82	Getting up, dressing, personal hygiene, toileting	4x
Lina	Rural	85	Medicine, dressing, putting on stockings, showering	3x

### Codes and categories

The analysis resulted in two main categories:

1. Mental presence
2. Having a say

The mental presence category emphasises the ability of health workers to act congruently and calmly, whereas having a say describes experiences of autonomy in patients’ everyday activities and how health workers facilitate such autonomy and pay attention to patients’ needs for closeness or distance. Table 3 shows the path from patients’ comments to the formation of categories and themes.

**Table 3:** From meaning units to themes [35]

Examples of meaning units, condensed meaning units, sub-themes, and themes from the analysis of interviews with older home care patients in Norway			
Meaning units	Condensed meaning units	Sub-themes	Themes
‘They’re supposed to be looking after me, not just acting selfishly and looking at their watch’. ‘They should be focusing on me, that’s actually their job’. ‘I wish they would sit down a bit, so I can see they’re relaxed’.	Correspondence between body language and actions Time for each individual patient Person-centred care	Congruent communication Keeping calm on a busy day	**Mental presence**
‘I want to decide when to go to bed myself, but they come and help me when they can’. ‘I can soon tell if there’s no contact. I just wish there were more people of the type I have real contact with!’	A desire to be independent, but still dependent on help Patients relating differently to different health workers	Autonomy Closeness and distance	**Having a say**

### Mental presence

This study highlights the importance of health workers’ ability to focus on patients by demonstrating that they are mentally present during the time they spend with patients. Congruent communication and the ability to act calmly despite being busy are factors that affect health worker-patient relationships. Patients felt that the health workers were mentally present when they engaged in small talk, were relaxed, and showed interest.

### Congruent communication

Correspondence between the health workers’ verbal and non-verbal language is emphasised as essential to the relationship. Congruence implies that there is no gap or conflict between what is said and how it is expressed. This is related to feelings of security and trust. Patients expected health workers to act in a genuine and credible manner.

Two participants said:

‘She should not act selfish by looking at her watch and saying, “I’ll have to leave soon”, since she’s supposed to be looking after me’*.* (Kristin)

‘They should be focusing on me, that’s actually their job’. (Kåre)

Even when health workers appear to act in a friendly manner, patients still sense whether they are mentally present or not, or have time for them. One informant said:

‘I can put up with a lot of things, but what I can’t stand is their phone ringing four times when they’re with me, so they have to break off and leave because then I feel like a parcel’. (Lina)

Patients find it objectionable and unpleasant when health workers’ phones keep ringing when they are being attended to.

### Keeping calm on a busy day

Being a health worker in an HCS involves visiting many patients daily. The day often seems hectic for both the health workers and patients. The patients in this study felt that the health workers had busy days, but they understood the situation. Two informants said:

‘Sometimes they’re busy; it’s really sad to see that’. (Thea)

‘What I miss is having more time with them, but they can’t really decide about their time themselves’. (Grete)

Some health workers are better than others at using their interpersonal skills to show that they have time for patients. Right from health workers’ initial greeting at the front door to other ways such as through their voices, body language, movements, and eye contact, patients can judge how busy they are, and they get a feeling of either calmness or busyness. However, some health workers may be busy without showing it to their patients. Two participants put it in this way:

‘It’s all about taking some time … not just running out of the door … they have to see things before they go’. (Anne)

*‘*I immediately realise what the visit will be like, and when I hear the voice in the hall, I know who it is’. (Thea)

Health workers use their time differently, with some being attentive to whether patients are comfortable before moving on. Since patients prefer health workers who show that they have time, time is linked to congruent communication:

‘I have the impression that they’re trained to talk to patients a bit about non-medical things as well, and make it a nice pleasant little visit’. (Grete)

Health workers who act in a calm and genuine manner are perceived as being mentally present.

### Having a say

Patients desire to have health workers who will help them become autonomous. They long to be independent, while also being dependent on help. This asymmetrical relationship disturbs the balance of power in the care situation. Patients are affected by the lack of complete control over their lives, even in their own homes.

### Autonomy

Patients want to be as independent as possible, and some health workers have interpersonal skills that enable them to meet patients’ needs for autonomy. The participants described how most health workers take the time to let them carry out and master tasks by themselves:

‘I try to put on my own clothes as much as I can, and enjoy doing it by myself, and they understand that very well and let me do it’. (Lina)

However, some participants described a lack of autonomy. Anne (76 years old) complained that she was not allowed to decide her own bedtime, and although she felt it was too early to go to bed and wanted to see the end of a film, she had to go to bed when the night worker came to help her. She objected to not being allowed to decide when to go to bed and was trying to maintain her autonomy:

‘I applied to get help to go to bed later, to which they agreed, but after only a few weeks, they started coming earlier again. I want to decide when to go to bed since I’m used to staying up late. Once when I wasn’t allowed to stay awake by the nurse, and told to go to bed, I decided to sit up all night instead. I’m not a child’. (Anne)

### Closeness and distance

The way Norwegian HCS are organized, patients rarely know the health worker who is likely to come on the next visit, and they often have to relate to a number of different health workers. This study shows that patients have different views on this, but only two of the participants described it as negative. The others thought it was a good arrangement, since they liked to meet different health workers:

‘There aren’t too many staff, and since they give me different things, I’m happy with the arrangement. I think it’s nice’. (Thea)

Some patients only seek pleasant interactions with health workers. Others want closer relationships. Lina, aged 85 years, who related to many people during her active professional life, favoured a critical approach relating to which health worker is assigned to whom, and believed that the optimal arrangement would be to match health workers and patients based on how well they fit together:

‘I want the home care leaders to consider each patient’s needs and then select a health worker whom they feel will do the patient good and be an inspiration’. (Lina)

This patient assessed whether she could have personal conversations with each health worker. She ‘chose’ them according to her needs. With some, she chatted about simple everyday things, while with others, she had deeper conversations, depending on the closeness of the relationship. She considered it just as nice to talk to young health workers, who, because of their fresh perspective, could teach her new things and offer different insights. Most patients adopted the same approach as Lina by assessing the health worker, the contact, and the relationship, and then making an independent decision about how close they wanted their relationship to be:

‘I can soon tell if there’s no contact. How I wish there were more people of the type that I have real contact with’! (Lina)

A close relationship can thus be established with some health workers, while there is more distance in the relationships with others.

## Discussion

We asked the following research question: What interpersonal skills do health workers need to establish a good relationship with older home care patients? A patient-oriented relationship is based on joint participation between health workers and patients [6]. As any interaction requires two people, both persons will influence the relationship. In the intersubjective field, the thoughts, feelings, or intentions of others are read and understood [23]. This study shows that when health workers are (1) mentally present in interactions, (2) congruent in communication, (3) calm and composed despite being busy, and (4) ensure patient autonomy, patients receiving HCS have a positive view of the relationship. This can be compared with Duffy et al.’s [21] descriptions of Important elements of interpersonal skills. In their point of view, the elements include treating others as one would want to be treated—with respect, paying attention to the patient, being mindful and personally present in the moment with the patient, having a caring intent, and being curious and interested in the patient’s life. It can also resonate well with what Bjornsdottir [15] claims in her study, “I try to make a net around each patient”. She describes home care nursing as relational and captures the idea of creating a net and the thoughts of providing time in home care, to develop understanding of each situation and each patients need.

A greater variety of tasks and more severely ill patients increase the pressure on health workers in HCS [10], who may find that they have insufficient time to fulfil patients’ needs for contact. This study shows that some health workers seemed to have more time than others, and although this is probably untrue, health workers’ interpersonal skills can provide calm and relaxed moments, despite their busy schedules. During patient visits, health workers give out signals that patients interpret. Their non-verbal actions—quick and hard movements, lack of eye contact, talking with their backs turned, and frequent glances at their watch—can give patients the impression that health workers have little time for them. Thus, incongruent messages will be dominated by such non-verbal language, which can explain as well as influence relationships. While time is not necessarily a vital factor, what is crucial is showing genuine interest and attention by being aware of the situation, making eye contact, asking questions, listening, and showing a gentle touch. Thus, if health workers demonstrate that they care, the number of minutes actually available is of less importance.

According to Rogers [36], congruence is one of the most important skills in interpersonal communication. Congruence in communication is important for relationships and can be experienced in just a few seconds [23]. Thus, even during short visits, health workers can make patients feel valued, if congruence and calmness are present in interactions. If the attitude of health workers is to be mentally present in the moment, observe and listen to patients, they will be perceived as congruent and genuine. It is during such moments of meeting [23], or opportunities, that health workers can demonstrate mental presence.

In Norway, HCS are organized in a way wherein patients who receive daily visits must relate to many different health workers [37, 38]. The reasons for this may be standardisation, rationalisation, and access to resources [37]. The present findings show that some patients are pleased to have several people to relate to, as also seen in other studies [39]. While some patients seek simple, friendly interactions that include small talk, others want deep and close relationships, turning to those health workers who are able to meet their needs. Consequently, health workers should be capable of realising each patient’s needs for closeness or distance.

This study shows the importance of interpersonal skills in ensuring patient autonomy. When people experience autonomy in a relationship with others, the relationship takes on a higher quality, which includes a feeling of security and psychological satisfaction [40]. The possibility of ensuring patient autonomy varies, but, in general, health workers can use their interpersonal skills to achieve this goal. The present findings are in accordance with the literature by showing that patients living at home want to have a say in structuring their daily lives and care [8], and also want to be involved in decision-making as far as possible [9, 12].

PCC and the need for autonomy will be achieved when patients encounter health workers with interpersonal skills, which allows them to make their own choices [6, 27–29]. Patients are not happy about being controlled and left out of decision-making, like the patient in this study who was not allowed to decide her own bedtime. Organisational factors—time and resources—will constrain health workers’ ability to ensure autonomy in HCS, despite the fact that autonomy is highly valued in today’s healthcare scenario [9]. However, health workers’ interpersonal skills will make them act differently in relation to meeting patients’ needs for autonomy.

Previous studies have shown that patients receiving HCS desire the fulfilment of their relational needs, rather than merely their physical and functional needs [41]. Many older patients are lonely and have a greater need for human contact [42, 43], and this is particularly relevant to home care recipients. Many lose their life partners in old age, while they themselves become physically weaker. Relationships with others are of great importance for a person’s health and the experience of well-being and meaning [44]. Health workers’ interpersonal skills are crucial, given that they are often the only persons with whom patients have contact the entire day [12]. At the same time, since patients differ and have varying needs, health workers need interpersonal skills to assess individual patients and meet their particular needs.

### Strengths and limitations

A strength of this study was that the interviews took place in the participants’ homes, in familiar and comfortable surroundings. Although there is no golden rule for the number of interviewees [45], since only 10 respondents formed the basis for this study, it could be a limitation. However, the number of participants enabled the researcher to produce detailed descriptions [46], based on thorough work throughout the interview process as well as in the analysis and interpretation of the phenomenon, thus using a ‘less can be more’ approach [45].

## Conclusions

Patients’ participation in decision-making is one of the key elements of modern healthcare. Hence, it is important to include patients’ perspectives in nursing education, which also involves providing PCC. This study sheds light on patients’ perspectives on the interpersonal skills needed by health workers to enhance their relationship with older home care patients. The foundation for a good health worker-patient relationship is laid when health workers are mentally present in the moment, remain calm on busy days, and facilitate patients’ autonomy. The time actually spent with patients is subordinate to the effect on patients of being cared for by mentally present and congruent health workers. Mental presence can create feelings of genuineness and security in relationships. Health workers often have hectic work days owing to workloads that make it difficult for them to find time to optimally perform their duties. Health workers who can facilitate patient autonomy and participation will increase patient satisfaction. Further, they need to develop an awareness of what each patient expects from the relationship: while some patients seek close relationships with health workers, which may include deep conversations, others are content with a less close relationship with pleasant small talk. No two patients are the same, and health workers should have the ability to adapt to the needs of individual patients.

## Data Availability

The dataset used during the current study are available from the corresponding author on reasonable request.

## Abbreviations

PCC: Person-centred care
HCS: Home care services

## References

[1] Markey K, O’Brien B, O’Donnell C, Martin C, Murphy J (2021). Enhancing undergraduate nursing curricula to cultivate person-centred care for culturally and linguistically diverse older people. Nurse Educ Pract.

[2] Stein-Parbury J. Patient & person: interpersonal skills in nursing. 6th ed: Elsevier; 2018.

[3] Bullington J, Söderlund M, Bos Sparén E, Kneck Å, Omérov P, Cronqvist A (2019). Communication skills in nursing: a phenomenologically-based communication training approach. Nurse Educ Pract.

[4] Fazio S, Pace D, Flinner J, Kallmyer B (2018). The fundamentals of person-centered care for individuals with dementia. Gerontologist.

[5] Strandås M, Bondas T (2018). The nurse-patient relationship as a story of health enhancement in community care: a meta-ethnography. J Adv Nurs.

[6] Suikkala A, Koskinen S, Katajisto J, Leino-Kilpi H (2020). Congruence between nursing students’ and patients’ views of student-patient relationships. Adv Health Sci Educ Theory Pract.

[7] Vabø M (2018). Omsorgsarbeid i et hverdagslivsperspektiv [Care work in an everyday life perspective]. Tidsskr Omsorgsforskning.

[8] Jacobs G (2018). Patient autonomy in home care: nurses’ relational practices of responsibility. Nurs Ethics.

[9] Breitholtz A, Snellman I, Fagerberg I (2013). Living with uncertainty: older persons’ lived experience of making independent decisions over time. Nurs Res Pract.

[10] Bing-Jonsson PC, Foss C, Bjørk IT (2015). The competence gap in community care: imbalance between expected and actual nursing staff competence. Nord J Nurs Res.

[11] Brennan JP (2020). I’d prefer to stay at home but I don’t have a choice’: Irish social workers’ experiences of decision-making in care planning with older people with dementia. Int Psychogeriatr.

[12] Dostálová V, Bártová A, Bláhová H, Holmerová I (2020). The needs of older people receiving home care: a scoping review. Aging Clin Exp Res.

[13] Strandås M, Wackerhausen S, Bondas T (2019). The nurse-patient relationship in the new public management era, in public home care: a focused ethnography. J Adv Nurs.

[14] Redjem R, Marcon E (2015). Operations management in the home care services: a heuristic for the caregivers’ routing problem. Flex Serv Manuf J.

[15] Bjornsdottir K (2018). ‘I try to make a net around each patient’: home care nursing as relational practice. Scand J Caring Sci.

[16] Glomsås HS, Knutsen IR, Fossum M, Halvorsen K (2020). User involvement in the implementation of welfare technology in home care services: the experience of health professionals - a qualitative study. J Clin Nurs.

[17] Norwegian Directorate of Health. Hjemmesykepleie og hjemmehjelp [home nursing and home help]: Helsenorge; 2019. https://www.helsenorge.no/hjelpetilbud-i-kommunene/helsetjenester-i-hjemmet/

[18] Bailey V (2007). Satisfaction levels with a community night nursing service. Nurs Stand.

[19] Rathert C, Williams ES, McCaughey D, Ishqaidef G (2015). Patient perceptions of patient-centred care: empirical test of a theoretical model. Health Expt.

[20] Daatland SO (2014). Boliggjøring av eldreomsorgen? [Senior care in the home?] (NOVA Report No. 24). Norsk Institutt for forskning om oppvekst, velferd og aldring/Norwegian Social Research.

[21] Duffy FD, Gordon GH, Whelan G, Cole-Kelly K, Frankel R, Buffone N (2004). Assessing competence in communication and interpersonal skills: the Kalamazoo II report. Acad Med.

[22] Peplau HE. Interpersonal relations in nursing: a conceptual frame of reference for psychodynamic nursing: Springer Publishing Company; 2004.

[23] Stern DN. The present moment in psychotherapy and everyday life: W.W. Norton; 2004.

[24] Thoresen L, Rugseth G, Bondevik H. Fenomenologi i helsefaglig forskning [phenomenology in health research]: Universitetsforlaget; 2020.

[25] Zangão MO, Mendes FRP (2015). Relational skills and preserving patient privacy in the caring process. Rev Bras Enferm.

[26] Schibbye ALL. Relasjoner: et dialektisk perspektiv på eksistensiell og psykodynamisk psykoterapi [relationships: a dialectical perspective on existential and psychodynamic psychotherapy]. 2nd ed: Universitetsforlaget; 2012.

[27] Diseth Å. Motivasjonspsykologi: hvordan behov, tanker og emosjoner fremmer prestasjoner og mestring [motivational psychology: how needs, thoughts and emotions promote achievement and mastery]: Gyldendal; 2019.

[28] Deci EL, Ryan RM. Intrinsic motivation and self-determination in human behavior: Springer; 1985.

[29] Mol A. The logic of care: Routledge; 2008.

[30] Bryman A. Social research methods. 5th ed: Oxford University Press; 2016.

[31] Fangen K. Deltagende observasjon [Participant observation]. 2nd ed: Fagbokforlaget; 2010.

[32] Heidegger M. Being and time: Basil Blackwell; 1962.

[33] Lincoln YS, Guba EG (1985). Naturalistic inquiry.

[34] Moser A, Korstjens I (2018). Series: practical guidance to qualitative research. Part 3: sampling, data collection and analysis. Eur J Gen Pract.

[35] From I, Leksell J, Marusarz M, Røli K, Sairanen R, Schrøder M (2009). Good nursing care for older nursing home clients in a Nordic context. Vård Nord.

[36] Rogers CR. Client-centered therapy: its current practice, implications, and theory: Constable; 1951.

[37] Gjevjon ELR (2015). Kontinuitet i hjemmesykepleien – vanskelige vilkår, men gode muligheter [Continuity in home nursing: difficult conditions, but good opportunities]. Tidsskr Omsorgsforskning.

[38] Kattouw CE, Wiig S (2018). Organiseringen av hjemmesykepleien kan gå ut over sikkerhet og kvalitet [the organization of home nursing can compromise safety and quality]. Sykepl Forskning.

[39] Widar M, Ek AC, Ahlström G (2007). Caring and uncaring experiences as narrated by persons with long-term pain after a stroke. Scand J Caring Sci.

[40] Weinstein N. Human motivation and interpersonal relationships: theory, research, and applications: Springer; 2014.

[41] Hellstrom Y, Persson G, Hallberg IR (2004). Quality of life and symptoms among older people living at home. J Adv Nurs.

[42] Due TD, Sandholdt H, Waldorff FB (2017). Social relations and loneliness among older patients consulting their general practitioner. Dan Med J.

[43] Jarling A, Rydström I, Ernsth-Bravell M, Nyström M, Dalheim-Englund AC (2018). Becoming a guest in your own home: home care in Sweden from the perspective of older people with multimorbidities. Int J Older People Nursing.

[44] Savikko N, Routasalo P, Tilvis RS, Strandberg TE, Pitkälä KH (2005). Predictors and subjective causes of loneliness in an aged population. Arch Gerontol Geriatr.

[45] Kvale S, Brinkmann S. Det kvalitative forskningsintervju [the qualitative research interview]. 3rd ed: Gyldendal akademisk; 2018.

[46] Geertz C. The interpretation of cultures: selected essays (Vol. 5043): Basic Books; 1973.

